# Supplementary material for: DMRforPairs: identifying Differentially Methylated Regions between unique samples using array based methylation profiles
Source: BMC Bioinformatics. 2014 May 15;15:141. doi: 10.1186/1471-2105-15-141 (PMC4046028; doi:10.1186/1471-2105-15-141)
Supplement: Additional file 2 — DMRforPairs output for the comparison of A431-MCF7 and NA17018-NA17105. Please start from the HTML files in each folder. Available via the BMC Bioinformatics website. [file 1471-2105-15-141-S2.zip › 1394847754114233_MOESM2_ESM/A431_MCF7/figures/7184.pdf]

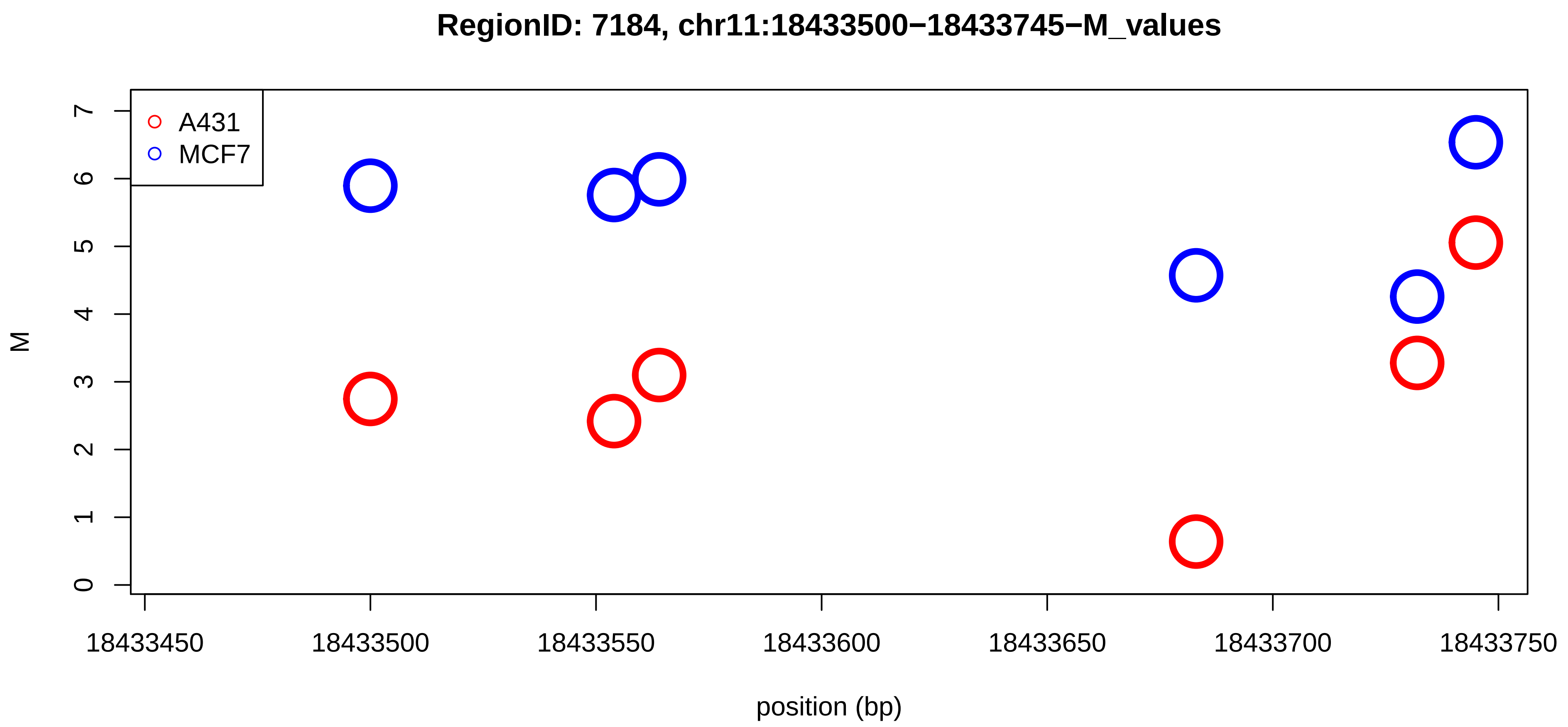
RegionID: 7184, chr11:18433500−18433745−M_values
A431
MCF7
M
0
1
2
3
4
5
6
7
18433450
18433500
18433550
18433600
18433650
18433700
18433750
position (bp)

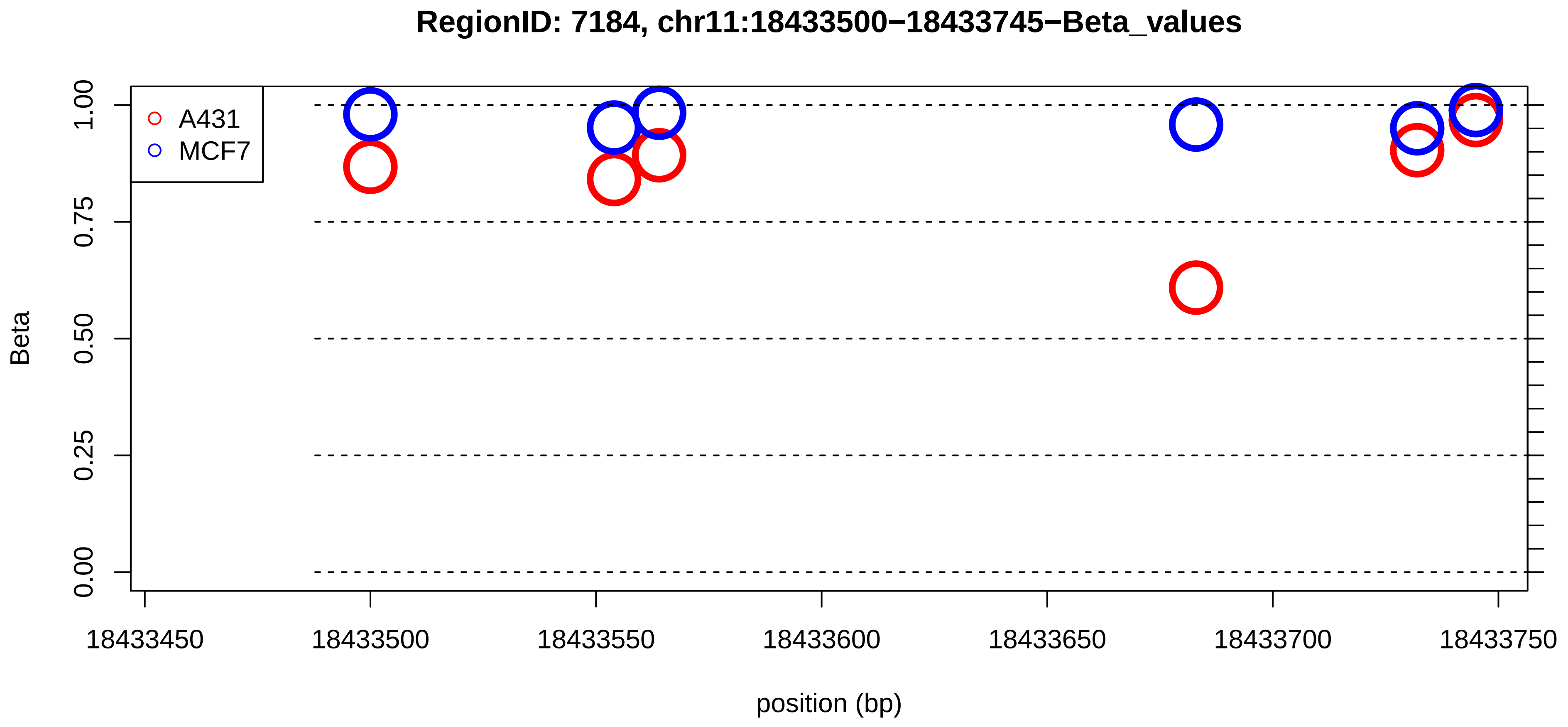
RegionID: 7184, chr11:18433500−18433745−Beta_values
A431
MCF7
Beta
0.00
0.25
0.50
0.75
1.00
18433450
18433500
18433550
18433600
18433650
18433700
18433750
position (bp)

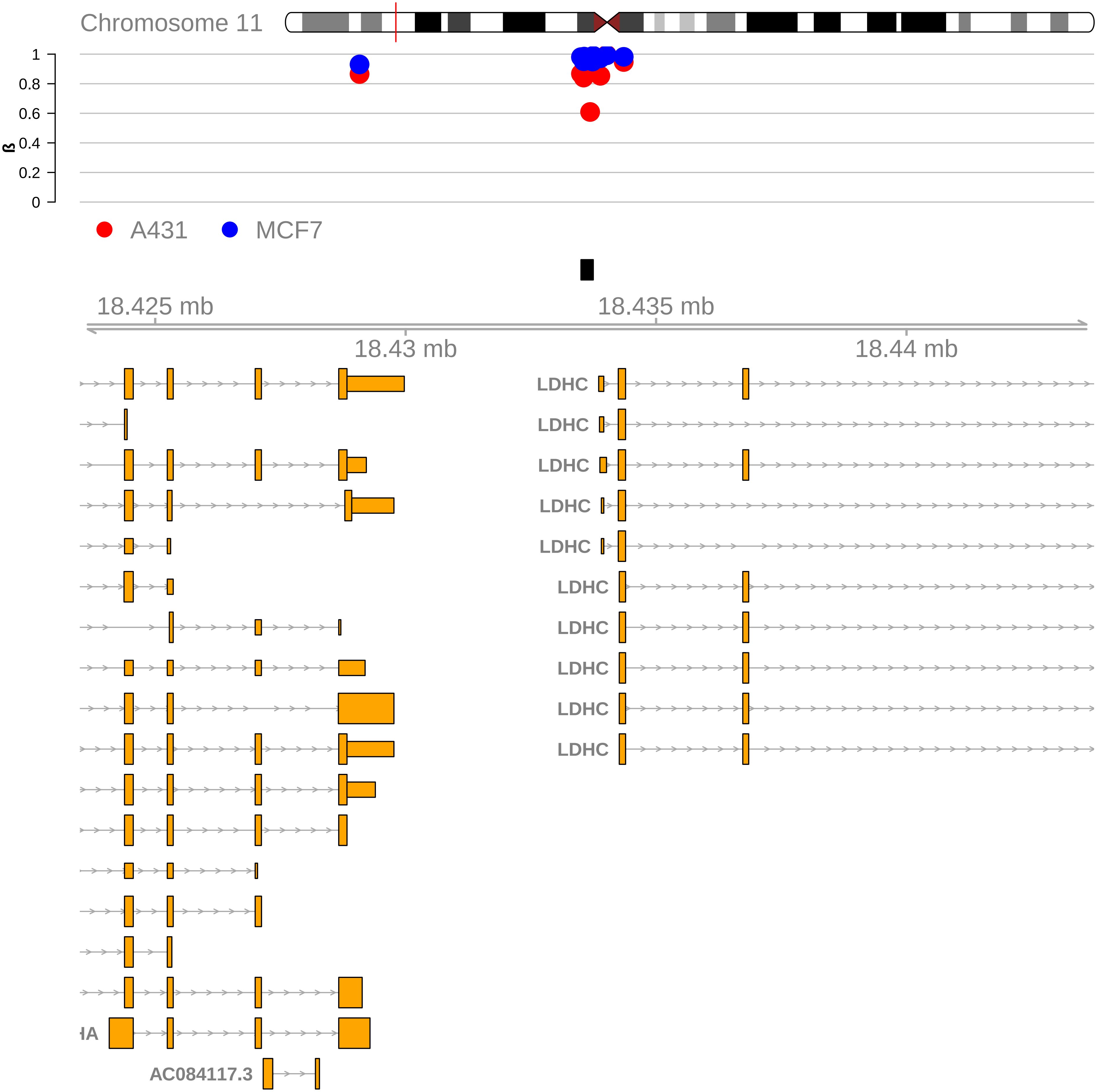

Chromosome 11
1
0.8
0.6
ß 0.4
0.2
0
A431
MCF7
18.425 mb
18.435 mb
18.43 mb
18.44 mb
LDHC
LDHC
LDHC
LDHC
LDHC
LDHC
LDHC
LDHC
LDHC
LDHC
AC084117.3
